# Supplementary material for: Crystal structure of inhibitor-bound human MSPL that can activate high pathogenic avian influenza
Source: Life Sci Alliance. 2021 Apr 5;4(6):e202000849. doi: 10.26508/lsa.202000849 (PMC8046417; doi:10.26508/lsa.202000849)
Supplement: Supplementary file 1 [file LSA-2020-00849_TableS1.docx]

**Table S1. Data collection and model refinement statistics.**

|  | Human MSPL (193-563)/decanoyl-RVKR-cmk |
| --- | --- |
| ***Data Collection*** ^a^ |  |
| X-ray source | PF-AR NE3A |
| Space group | *P*2_1_2_1_2_1_ |
| Unit cell parameters | *a* = 55.84 Å, *b* =62.40 Å, *c* = 171.63 Å,  α = 90°, β = 90°, γ = 90° |
| Wavelength, Å | 1.0000 |
| Resolution range, Å | 40-2.6 (2.72-2.60) |
| No. observed reflections | 130,814 |
| No. unique reflections | 19,086 |
| Multiplicity | 6.9 (7.0) |
| Completeness, % | 99.7 (99.6) |
| < *I* >/<σ (*I*) > | 9.5 (2.5) |
| *CC* _1/2_ | 0.995 (0.729) |
| *R*_merge_ ^b^ | 0.151 (1.012) |
| ***Model Refinement*** |  |
| Resolution range, Å | 40-2.6 |
| No. reflections | 17,570 |
| *R*_work_ / *R*_free_ ^c^ | 0.184 / 0.235 |
| No. non-H atoms |  |
| Protein | 2,911 |
| Oligosaccharide | 52 |
| Inhibitor | 50 |
| Ion/water | 36/81 |
| Average *B*-factors, Å^2^ |  |
| Protein | 35.1 |
| Oligosaccharide | 58.5 |
| Inhibitor | 41.3 |
| Ion/water | 58.0/26.2 |
| r.m.s deviations |  |
| Bond lengths, Å | 0.004 |
| Bond angles, ° | 1. 191 |
| Ramachandran plot ^d^, % |  |
| Favored region | 94.3 |
| Allowed region | 5.7 |
| Outlier region | 0.0 |
| PDB entry | 6KD5 |

^a^ Highest resolution shell is shown in parentheses.

^b^ *R*_merge_ = Σ*_hkl_* |*I_i_* - <*I_i_*>| / Σ*_hkl_* *I_i_*, where *I_i_*(*hkl*) is the intensity of the *i*th measurement of reflection *hkl* and <*I_i_*(*hkl*)> is the average value of *I_i_*(hkl) for all *i* measurements.

^c^ *R*_work_ = Σ*_hkl_* ||*F*_obs_| - |*F*_calc_|| / Σ*_hkl_* |*F*_obs_|. 8% of the reflections were excluded for *R*_free_ calculation.

^d^ Analyzed with the program *Rampage* (Lovell et al, 2003).

# Reference

Lovell SC, Davis IW, Arendall WB 3rd, de Bakker PI, Word JM, Prisant MG, Richardson JS, Richardson DC (2003) Structure validation by Cα geometry: Φ, ψ and cβ deviation. *Proteins* 50: 437–450. 10.1002/prot.10286
